# Association between social networks and discussions regarding advance care planning among Japanese older adults

**DOI:** 10.1371/journal.pone.0213894

**Published:** 2019-03-25

**Authors:** Jun Miyashita, Yosuke Yamamoto, Sayaka Shimizu, Takuya Aoki, Teruhisa Azuma, Toshihiko Takada, Michio Hayashi, Miho Kimachi, Tatsuyoshi Ikenoue, Shingo Fukuma, Shunichi Fukuhara

**Affiliations:** 1 Department of Healthcare Epidemiology, School of Public Health in the Graduate School of Medicine, Kyoto University, Kyoto, Japan; 2 Department of General Medicine, Shirakawa Satellite for Teaching and Research (STAR), Fukushima Medical University, Fukushima, Japan; 3 Human Health Sciences in the Graduate School of Medicine, Kyoto University, Kyoto, Japan; 4 Center for Innovative Research for Communities and Clinical Excellence (CIRC2LE), Fukushima Medical University, Fukushima, Japan; Texas Technical University Health Sciences Center, UNITED STATES

## Abstract

**Background:**

Older adults’ discussions with family, or with physicians, or with both, about advance care planning (ACP) are increasingly regarded as important for the management of end-of-life care, and yet the factors that induce older adults to engage in ACP discussions are poorly understood. For example, in older adults, is stronger connectedness with family and friends (stronger “networks”) associated with ACP discussions? By facilitating, or by impeding ACP discussions? We sought to evaluate the associations between ACP discussions and social networks in Japanese older adults.

**Methods:**

In July 2016 we conducted a cross-sectional survey on 355 community-dwelling patients aged ≥65 years visiting community hospital clinics in Fukushima, Japan. We used the Lubben Social Network Scale (LSNS-6, the shortest available LSNS scale) to assess social networks and recorded two components of social network structure, marital status (dichotomized as “married” vs. “single / other”) and living status (“living with others” vs. “living alone”). One item asked if patients had had ACP discussions. We analyzed the LSNS-6 social network and marital and living status data in relation to the occurrence of ACP discussions using multiple logistic regression models with adjustments for possible confounding factors.

**Results:**

Respondents’ social network was “limited” in 16% of cases; 61% had had ACP discussions. Respondents with a limited social network had a significantly lower tendency to have had ACP discussions than respondents with an “adequate” social network (adjusted odds ratio [AOR]: 0.35; 95% confidence interval [CI]: 0.18–0.66; *P* < 0.001). Marital status and living status were not significantly associated with ACP discussion.

**Conclusions:**

Among Japanese older adults, weaker social networks may be associated with a lower tendency to discuss ACP. Our findings may help practitioners to quickly screen populations at risk for inadequate ACP discussion by using the LSNS-6.

## Introduction

Advance care planning (ACP) is defined as a process whereby patients, in consultation with healthcare providers, family members, and significant others, make important decisions about their future health care, especially end-of-life care [1]. The main focus of ACP has recently shifted from the completion of advance directives (ADs) to discussions about ACP among individuals, surrogates, and clinicians [2]. Previous studies have shown that patient-centered ACP discussions improve the quality of end-of-life care and reduce family emotional trauma and utilization of health care services [3–5].

Although the effectiveness of ACP discussions has been demonstrated, their prevalence varies widely by country. For example, while the prevalence of ACP discussions more than doubled among Japanese adults from 20% in 2006 to 42% in 2014 [6,7], it is still much lower than prevalences in the U.S. (67%) [8] and Canada (52%) [9]. In addition, although a guideline supporting patients’ decision-making for end-of-life care exists, Japan has neither legally established mandatory advance directives nor legislation supporting advance care planning, unlike the case of the Patient Self-Determination Act in the United States. Partly due to this, the prevalence of advance directives was reported to be very low (3%) in 2014 [7]. Previous studies have reported various sociodemographic factors related to the occurrence of ACP discussions: for example, age, sex [10], marital status [11,12], ethnicity [13], experience of past hospitalization [12], income [14], and education level [12]. Nevertheless, identification and a better understanding of factors associated with the occurrence of ACP discussions, which may include social relationships and their features, can reasonably be expected to facilitate and expand the implementation of ACP, even in countries with high prevalences.

A vast literature has shown that social relationships are important for human well-being and maintenance of health [15,16]. The term “social networks” refers here not to the burgeoning use of handheld telecommunications devices but to the web of affiliations that connect individuals to each other, and the characteristics of those ties are also important [17]. According to the conceptual model proposed by Berkman et al. [17], social networks influence health via such pathways as social support, social influence, social engagement, and access to resources. Social networks’ functional aspects, defined as the qualitative and behavioral aspects that underlie those pathways, are especially important [18]. Previous studies suggest that specific components of social network structure such as marital and parenthood status [10–12,19] might be associated with patients’ tendency to engage in ACP discussions. A study in Japan reported that Japanese older adults living alone had a lower (albeit not statistically significant) tendency to hold ACP discussions [20]. We are not aware, however, of any studies that focus on both the structural and the functional aspects of social networks in relation to older adults’ propensity for ACP discussion.

This study therefore sought to investigate whether both structural and functional features of social networks assessed among Japanese community-dwelling older adult patients by using the shorter Lubben Social Network Scale (LSNS-6) are associated with these patients’ propensity (or unwillingness) to hold ACP discussions. We sought to identify associations between having held ACP discussions and two specific social network structural characteristics, namely marital status (as the dichotomous categorical variable “(currently) married vs. single / other”) and living status (living with others vs. living alone).

## Materials and methods

### Study design, setting, and participants

This study adopted a cross-sectional design using a questionnaire survey. It was conducted in the outpatient department at Shirakawa Kosei General Hospital, a 471-bed community teaching hospital which provides medical services to Shirakawa city and the rural area surrounding it, home to 150,000 inhabitants in the southern part of Fukushima Prefecture, north of Tokyo. A self-administered questionnaire was distributed to all outpatients aged 65 and over who visited the outpatient departments of general internal medicine, gastroenterology, cardiology, endocrinology, respiratory medicine, or orthopedics during a one-week period in July 2016. The inclusion criterion was being a community-dwelling outpatient aged 65 years and over who was capable of responding and willing to respond to the questionnaire. Participants were informed in the explanation form before participating in this survey that they were under no obligation to answer any questions and they were able to rescind the consent to participate at any time. We also informed them that completing questionnaire was considered as consent to participate. This study was approved by the Ethics Committee of the Kyoto University Graduate School and Faculty of Medicine (R0270-2).

### Variables, measurements, and classification

#### Outcome

The response variable (outcome) was ACP discussion. Previous studies [12,21] conducted in the U.S. report that an approach combining both formal ADs and informal discussions is more effective than AD completion alone and that very few ADs are completed in the absence of informal ACP discussions. Considering these findings and the situation in Japan, where ADs have not been given formal legal sanction and are not in general use, we decided to define the response variable as the occurrence of ACP discussions, meaning informal exchanges of views about advance care planning with family member(s) or primary care doctor(s) or both. In accordance with practice in previous studies [9,13], we provided all patients with information about ACP, accompanied by the following instructions (in Japanese): “ACP is a process that makes known your wishes concerning the kind of health care you wish to receive or not to receive if you should become very ill or injured and be unable to speak for yourself. ACP is sometimes referred to as a ‘living will’ or an ‘advance directive’.” Respondents were then asked whether they had ever discussed with family members or a primary care doctor the health care treatment(s) they wanted to receive or would refuse if they became too ill or injured to speak for themselves.

#### Main exposures

Our aim was to evaluate the association between social networks and ACP discussions. We began by evaluating the respondents’ social network(s) using the Japanese validated LSNS-6, the shortest version of several available Lubben social network scales (See S1 Appendix) [22]. The LSNS-6 [23] was developed with a focus on close relationships, and can be used as an extremely quick measure to screen for social isolation in a clinical setting [24]. It assesses social ties by asking respondents three questions each about the number of family members and about the number of friends in their social network who they contact at least once a month (structure), who they can ask for help (tangible aid), and who they are comfortable with when talking about private matters (emotional support) [18]. The total scale score is an equally weighted sum of the six 5-step items, with total scores ranging from 0 to 30. Lubben et al. [23] assigned the cutoff point to scores of less than 12 out of 30 and considered these respondents at risk for social isolation. We accordingly defined respondents with scores of less than 12 as having a “limited” social network, and those with scores of 12 and over as having an “adequate” social network (Category-2 LSNS-6). We further divided respondents with a limited social network (Category-3 LSNS-6) into two subcategories—severely and mildly limited—based on the median value.

We then looked at marital status and living status to assess specific components of social network structure. Marital status was noted as married vs. single / divorced / widowed. Living status was noted as living alone vs. living with someone.

#### Confounding factors

We took note of socio-demographic characteristics (age, sex, educational level) and health characteristics (physical health status, mental health status). Age and sex have been repeatedly reported to influence ACP [10,11]. Educational status has also been reported to influence end-of-life preferences and the association between social relationships and ACP [11,12]. Educational level’s cutoff was set in a previous study at “12 years or less” or “more than 12 years” [12]. Physical health status and mental health status are among the general health status factors that have been used in the analyses of previous studies [13]. Physical health status was measured using the physical functioning subscale (PF) and mental health status was measured using the mental health subscale (MH) of the 12-Item Short Form Survey (SF-12), respectively. These scores were transformed with a mean of 50 and a standard deviation of 10 in the general Japanese population. The scores were then transformed into dichotomous variables with a cutoff score of 50 [25].

### Statistical analysis

We used Student’s *t* test or the chi-square test for bivariate analyses. To evaluate associations between social network categories and the occurrence of ACP discussions, we used three separate multiple logistic regression models. In the first model, the association between Category-2 LSNS-6 and the occurrence of ACP discussions was evaluated. In the second model, the association between Category-3 and the occurrence of ACP discussions was evaluated. In the third model, the association between specific components of social network structure, which included marital status and living status, and the occurrence of ACP discussions was evaluated. All multiple logistic regression models were adjusted for the following confounding factors: age, sex, educational level, physical health status, and mental health status. In addition, in the second model, a test of linear trends across Category-3 LSNS-6 was performed to evaluate the linearity of the relationship between social networks and occurrence of ACP discussions based on a previously reported method [26].

#### Missing values

We also performed a multiple imputation procedure using the chained equations method for 70 (19.7%) respondents with at least one missing data item, based on the assumption of missing at random. The marital status variable had the most missing data in the dataset (n = 40, 11.2%). The missing values were imputed using factors including the occurrence of ACP discussions, scores of LSNS-6, and other covariates. We created 20 imputed datasets and then employed logistic regression models for each dataset. The results derived from 20 models were integrated using Rubin’s rule. In addition, we performed a sensitivity analysis using listwise deletion. STATA/IC 14 (StataCorp, College Station, TX) was used for statistical analysis.

## Results

### Sociodemographic and health characteristics of the participants

Of the 809 patients aged 65 and over visiting the clinics, 355 responses were received (response rate: 43.9%). No significant differences were found between responders and non-responders in age (75.9 years vs. 76.5 years, *P* = 0.12) or sex (50.1% male vs. 49.3% male, *P* = 0.82). In total, 56 (16.1%) respondents were classified as having a limited social network. Significant differences between those with a limited social network and those with an adequate social network were found in mental health status by MH score of SF12 (43.8 with a standard deviation (SD) of 13.4 vs. 49.6 with a SD of 10.4) and in physical functioning by PF score of SF12 (29.5 with a SD of 19.9 vs. 37.6 with a SD of 18.3), as well as in number of ACP discussions (42.3% vs. 64.6%) (Table 1).

**Table 1:** Sociodemographic and health characteristics of the respondents.

	Total sample	Social networks by LSNS-6		*P*-values	Incomplete
		Limited	Adequate		
		(<12)	(> = 12)		
Sociodemographic and health factors	n = 355	n = 56	n = 292		7
Male (vs. Female), Number (%)	178 (50.1)	24 (42.9)	149 (51.0)	0.26^a^	0
Age (years), Mean (SD)	75.9 (7.58)	77.1 (8.7)	75.6 (7.4)	0.17^b^	0
Married (vs. Unmarried, divorced or widowed), Number (%)	215 (68.3)	29 (58.0)	182 (70.3)	0.09^a^	40
Educational level ≤12 years (vs. > 12), Number (%)	275 (83.6)	45 (84.9)	223 (82.9)	0.72^a^	26
Living with someone (vs. Living alone), Number (%)	292 (87.2)	43 (81.1)	242 (88.0)	0.175^a^	20
Physical health status by PF of SF12, Mean (SD)	36.2 (19.2)	29.5 (19.9)	37.6 (18.3)	0.002^b^	19
Mental health status by MH of SF12, Mean (SD)	48.6 (11.3)	43.8 (13.4)	49.6 (10.4)	<0.001^b^	23
Advance care planning discussions, Number (%)	195 (61.1)	22 (42.3)	170 (64.6)	0.003^a^	36

Abbreviations: LSNS-6, Lubben Social Network Scale; SF-12, the 12-Item Short Form Survey; PF, physical functioning subscale of SF-12; MH, mental health subscale of SF-12.

^a^Chi-square test

^b^Student’s *t* test

### Association between social networks and ACP on multivariable analysis

The respondents with a limited social network had a significantly lower tendency to hold ACP discussions than those with an adequate social network (adjusted odds ratio [AOR]: 0.35; 95% confidence interval [CI]: 0.18–0.66; *P* = 0.001) (Table 2).

**Table 2:** Association between social networks assessed by LSNS-6 and advance care planning discussions.

	AOR, 95%CI
Limited social networks, LSNS-6 scores <12 (vs. ≥12)	0.35, 0.18–0.66**
Age (per year)	1.02, 0.99–1.06
Male (vs. Female)	0.80, 0.50–1.29
Educational level ≤12 years (vs. >12 years)	0.89, 0.47–1.67
Low physical health status by PF of SF12, ≤50 (vs. >50)	1.29, 0.78–2.11
Low mental health status by MH of SF12, ≤50 (vs. >50)	1.28, 0.79–2.08

Abbreviations: AOR, adjusted odds ratio; 95%CI, 95% confidence interval; LSNS-6, Lubben Social Network Scale; SF-12, the 12-Item Short Form Survey; PF, physical functioning subscale of SF-12; MH, mental health subscale of SF-12.

*P < .05

**P < .001

**Fig 1:**
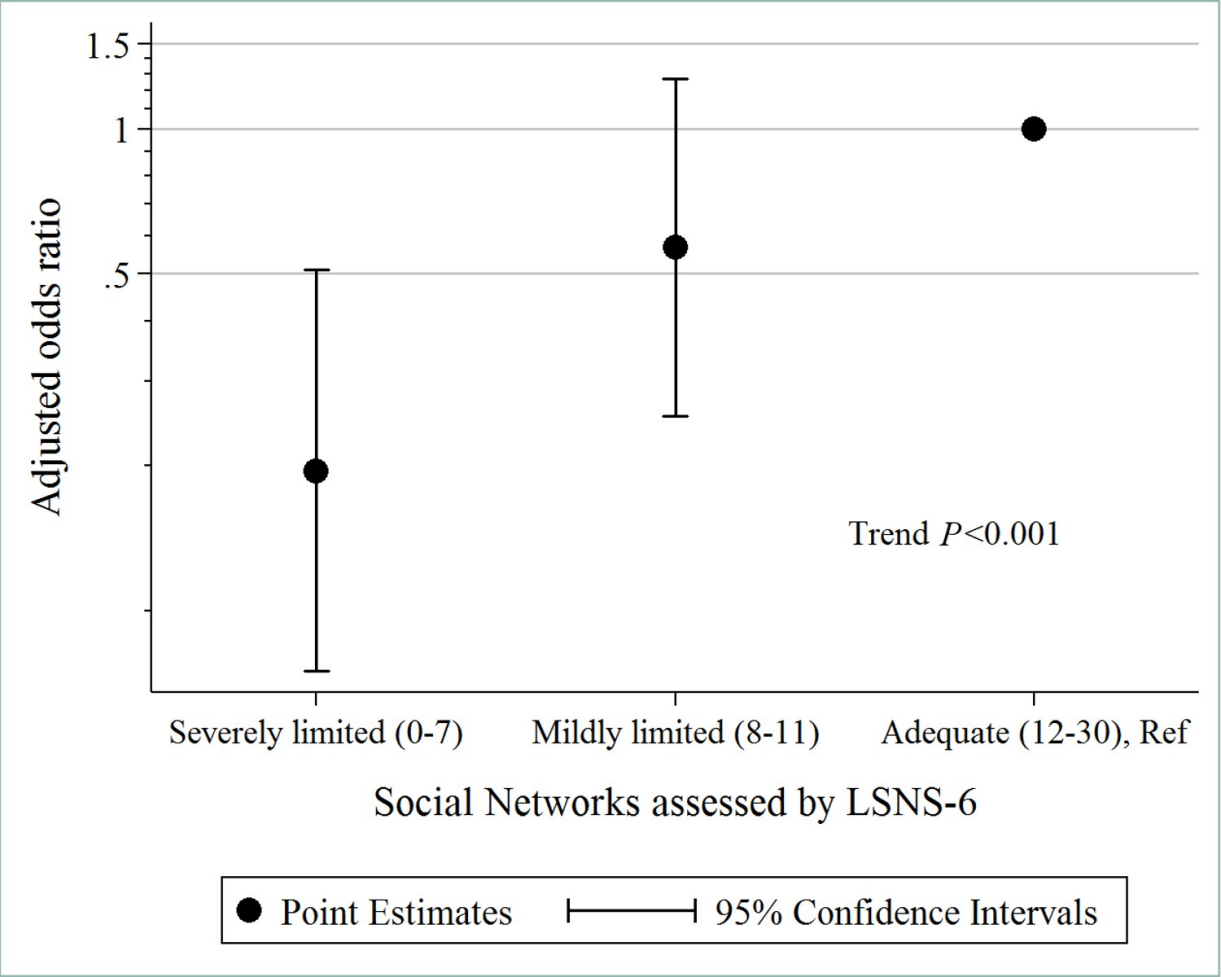
Odds ratio of occurrence of advance care planning discussion by Lubben Social Network Scale. Abbreviations: LSNS-6, Lubben Social Network Scale; Ref, Reference.

Compared with respondents with an adequate social network, respondents with a severely limited social network (LSNS-6, 0–7) and with a mildly limited social network (LSNS-6, 8–11) had a significantly lower tendency to hold ACP discussions, with AORs of 0.20 and 0.57, respectively (*P* for trend <0.001) (Fig 1).

No significant association was found between living status and ACP discussions, nor between marital status and occurrence of ACP discussions (Table 3).

**Table 3:** Association between social network structure and advance care planning discussions.

	AOR, 95%CI
Single, divorced, or widowed (vs. Married)	0.71, 0.37–1.34
Living alone (vs. Living with someone)	0.95, 0.43–2.09
Age (per year)	1.03, 0.99–1.06
Male (vs. Female)	0.77, 0.48–1.24
Educational level ≤12 years (vs. >12 years)	0.88, 0.47–1.65
Low physical health status by PF of SF12, ≤50 (vs. >50)	1.16, 0.71–1.88
Low mental health status by MH of SF12, ≤50 (vs. >50)	1.12, 0.70–1.80

Abbreviations: AOR, adjusted odds ratio; 95%CI, 95% confidence interval; SF-12, the 12-Item Short Form Survey; PF, physical functioning subscale of SF-12; MH, mental health subscale of SF-12.

In the sensitivity analysis using listwise deletion, all results were identical to those in the primary analysis with multiple imputation; limited social networks were significantly associated with occurrence of ACP discussions, whereas no significant associations were found between marital status or living status and occurrence of ACP discussions.

## Discussion

Much like other Asian countries, Japan has a long tradition of family-centered decision-making and places priority on beneficence rather than on autonomy in medical ethics [27–30]. Thus family-centered medical decisions are a function of filial piety; illness is considered a family matter as opposed to an individual one [31]. A previous qualitative study [32] conducted among Japanese and Japanese Americans reported that there was discordance between what Japanese and Japanese American adults would want done for themselves at the end of life (avoidance of "*meiwaku*," a Japanese word meaning "trouble for others") and what they felt compelled to do for family members ("*kazoku no jô*"); most Japanese and Japanese American adults have a tendency to eschew prolonged survival taking into account the burden on family caregivers, whereas their caregivers’ responsibility does not permit them to give up on a loved one. This discordance might impede the holding of ACP discussions (because the Japanese tend to dislike confrontations) but at the same time it might increase the need for older adults and their family to talk about ACP (because their avoidance of confrontation may later generate *meiwaku*). It is important to know how this discordance plays out in actual settings and what can be done to encourage timely discussions.

Previous studies conducted in the U.S. reported that older adults’ ACP discussions were associated with several of the psychosocial mechanisms in Berkman’s model, i.e. such social supports as general family functioning, marital satisfaction, emotional support from children [13,33], and social influences such as the family’s and significant others’ experiences of a painful death or of a good end of life [12,34]. The association between these various psychosocial mechanisms and ACP discussions may be confounded by other mechanisms, however, because they operate simultaneously and are not mutually exclusive [17]. We chose social networks, a simpler concept located in a more objective and macrosocial process in Berkman’s model, as the major exposure in this study because we judged that, in the clinical setting, it is most important first to screen individuals for their likelihood to engage in ACP discussion.

The present study found that inadequate (“limited”) social networks, assessed by using LSNS-6, are strongly associated with a less frequent occurrence of ACP discussions; in other words, the fewer people an older adult knows and sees and relies on, the less likely it is that ACP will take place (Table 2). Our study also found that the size of limited social networks was proportional to the occurrence of ACP discussions: Japanese older adults with severely limited social networks (LSNS scores 0–7) were even less likely to have had ACP discussions than Japanese older adults with only mildly limited social networks (LSNS scores 8–11) (Fig 1). The findings of this study therefore suggest that social networks that are functionally and structurally more adequate may in some way facilitate willingness among Japanese older adults to hold ACP discussions.

We found, however, that two specific structural aspects of social networks (marital and living status) were not significantly associated with occurrence of ACP discussions. This might be partly because of the small sample size; we nevertheless found that social network metrics assessed by using LSNS-6 had a stronger influence on ACP discussions than did social network structure alone. One plausible explanation is that what is more important for occurrence of ACP discussions among Japanese older adults might be not the structural but the functional aspects of social networks, in which diverse behavioral pathways are operative [17,18].

The pathways underlying the association between social networks and ACP discussions among Japanese older adults have not, to the best of our knowledge, been clarified. Social engagement might be one functional pathway that mediates between social networks and ACP discussions; evidence of this is found in several recent studies regarding the effect of social engagement on other health-promoting behaviors among Japanese older adults, such as dental hygiene or better glycemic control [35,36]. Social support is another, according to the aforementioned studies conducted in the U.S. [13,33]. However, it remains unclear whether perceived social support from family might affect ACP discussions positively or negatively in Japan, a family-centered decision-making society, where caregivers’ responsibilities for family members are strongly influenced by filial piety. Further studies in future will be required to detect which pathway(s) affect social networks and the occurrence of ACP discussions in Japanese older adults.

The strengths of this study are, first, that we for the first time established that the larger a Japanese older adult’s social network, evaluated in terms both of structure and function as the LSNS-6 does, the greater the Japanese older adult’s likelihood to engage in ACP discussions. Second, by using a convenient instrument, LSNS-6, we identified a possible predictor of the subpopulation of Japanese older adults who are at risk for failure to hold timely and adequate ACP discussions.

This study had several limitations. First, because all variables were self-reported, some measurement bias, such as misclassification or reporting bias, might have occurred, despite the similarities of the questionnaire used in this study to those used in previous studies [9,13,33]. Second, involvement of unknown or unmeasured confounding factors is possible. For example, having adult children is another structural aspect of social networks. Because the previous studies in the U.S. demonstrated no significant association between having adult children and ACP discussions [12,33], we did not measure this factor as a specific structural aspect of social networks. However, in Japanese culture where filial piety is valued, the result of the previous studies in the U.S. might not be identical to the result in the Japanese situation. Therefore, a further study will be needed to examine associations between having adult children and the occurrence of ACP discussions in Japan. Third, because the response rate in this study was less than half, our results might have been affected by nonresponse bias, although there were no differences in age or sex between responders and nonresponders. Finally, evidence for the generalizability of our findings to other regions in Japan is scant because this study was conducted in only one site, albeit presumably an “average” one because the hospital where this survey was conducted provides medical services to 150,000 inhabitants of a medium-sized city and its surrounding rural area.

In conclusion, larger social networks appear to facilitate, not impede, ACP discussions among Japanese older adults. Our results indicate that it is possible to detect the older adults with a limited social network that healthcare providers should prioritize to facilitate the ACP discussion process. To increase the rate of ACP implementation in Japan, it is important to develop an ACP promotion program with intensive interventions tailored for older adults with an inadequate social network.

## Supporting information

S1 Appendix Lubben Social Network Scale (LSNS-6). (DOCX)

S1 File Social networks and ACP.xls. (XLS)
