# Supplementary material for: Association between social networks and discussions regarding advance care planning among Japanese older adults
Source: PLoS One. 2019 Mar 25;14(3):e0213894. doi: 10.1371/journal.pone.0213894 (PMC6433343; doi:10.1371/journal.pone.0213894)
Supplement: S1 Appendix — (DOCX) [file pone.0213894.s001.docx]

**S1 Appendix. Lubben Social Network Scale (LSNS-6)**

FAMILY: Considering the people to whom you are related by birth, marriage, adoption, etc.

1. How many relatives do you see or hear from at least once a month?

0 = *none* 1 = *one* 2 = *two* 3 = *three or four* 4 = *five thru eight* 5 = *nine or more*

2. How many relatives do you feel close to such that you could call on them for help?

0 = *none* 1 = *one* 2 = *two* 3 = *three or four* 4 = *five thru eight* 5 = *nine or more*

3. How many relatives do you feel at ease with that you can talk about private matters?

0 = *none* 1 = *one* 2 = *two* 3 = *three or four* 4 = *five thru eight* 5 = *nine or more*

FRIENDSHIPS: Considering all of your friends including those who live in your neighborhood

4. How many of your friends do you see or hear from at least once a month?

0 = *none* 1 = *one* 2 = *two* 3 = *three or four* 4 = *five thru eight* 5 = *nine or more*

5. How many friends do you feel close to such that you could call on them for help?

0 = *none* 1 = *one* 2 = *two* 3 = *three or four* 4 = *five thru eight* 5 = *nine or more*

6. How many friends do you feel at ease with that you can talk about private matters?

0 = *none* 1 = *one* 2 = *two* 3 = *three or four* 4 = *five thru eight* 5 = *nine or more*
